# Loss of Trop2 causes ErbB3 activation through a neuregulin-1-dependent mechanism in the mesenchymal subtype of HNSCC

**DOI:** 10.18632/oncotarget.2423

**Published:** 2014-09-02

**Authors:** Kaihua Zhang, Lamont Jones, Sora Lim, Christopher A. Maher, Douglas Adkins, James Lewis, Randall J. Kimple, Elana J. Fertig, Christine H. Chung, Andreas Herrlich, Matthew J. Ellis, Brian A. Van Tine, Loren S. Michel

**Affiliations:** ^1^ Division of Oncology, Department of Internal Medicine, Washington University School of Medicine, St. Louis, Missouri; ^2^ The Alvin J. Siteman Cancer Center, Washington University School of Medicine, St. Louis, Missouri; ^3^ Department of Pathology, Washington University School of Medicine, St. Louis, Missouri; ^4^ Department of Human Oncology, University of Wisconsin, Madison, Wisconsin; ^5^ Department of Oncology, The Johns Hopkins University School of Medicine, Baltimore, Maryland; ^6^ Renal Division, Brigham and Women's Hospital, Harvard Medical School, Boston, Massachusetts

**Keywords:** Trop2, ErbB3, Head and Neck Squamous Cell Carcinoma (HNSCC), Neuregulin-1, Therapy

## Abstract

In head and neck squamous cell cancer (HNSCC), four intrinsic subtypes (or groups) have been identified, and each one possesses a unique biology that will require specific treatment strategies. We previously reported that mesenchymal (group 2) tumors exhibit reduced levels of Trop2 expression. In this study, we investigated the functional role of Trop2 in HNSCC and find that loss results in autocrine activation of the EGFR family member ErbB3 via neuregulin-1. Trop2 localizes to both the cell surface and cytosol of HNSCC cells and forms a complex with neuregulin-1, which is predominantly cytosolic. Inactivation of Trop2 increases the concentration of neuregulin-1 at the cell surface where it is cleaved to activate ErbB3. In primary HNSCC, detection of ErbB3 activation was limited to Trop2 negative tumors. An analysis of the Cancer Genome Atlas (TCGA) HNSCC dataset confirms enrichment for ErbB3 activity in mesenchymal tumors. Notably, Trop2 loss triggers sensitivity to anti-ErbB3 antibodies, which results in reduced proliferation and tumorigenic growth of Trop2 negative HNSCC cancer cells. These results uncover a molecular mechanism by which tumor cells control the amount of cell-surface neuregulin-1 available for cleavage and ErbB3 activation. Moreover, we demonstrate that Trop2 is a potential surrogate biomarker to identify tumors with ErbB3 activation and may therefore respond to anti-ErbB3 therapeutics.

## INTRODUCTION

Head and neck squamous cell carcinoma (HNSCC) is a highly heterogeneous disease whose behavior is dictated by the site of origin within the head and neck as well as the underlying etiology [1]. Molecular profiling of these biologically heterogeneous tumors has revealed four distinct subtypes [2]. Recently this classification was validated by Walter, et al. and The Cancer Genome Atlas (TCGA) Project in two independent tumor dataset [3, 4], and the nomenclature has now been updated to reflect their biology: basal (Group 1), mesenchymal (Group 2), atypical (Group 3) and classical (Group 4) [3]. HNSCC subtypes have been found to possess unique biological features that are associated with very different outcomes. For example, basal tumors were reported to exhibit high EGFR pathway activity, while mesenchymal tumors, which comprise approximately twenty-five percent of HNSCC, exhibited low EGFR activity; the atypical subtype was enriched in human papillomavirus-related HNSCC; and the classical subtype was shown to be enriched for genes associated with exposure to cigarette smoke [2, 3]. Despite the recent validation of the HNSCC subtypes, much remains to be learned concerning their biology before knowledge of the subtypes will have an impact on clinical decision-making. This situation lies in stark contrast to the successful application of subtype information in other tumor types such as breast [5] and colon [6] cancer, where molecular subtyping guides the selection of targeted therapies as well as the rational development of experimental therapeutics.

We recently reported that expression of Trop2, a transmembrane protein that is emerging as a pleiotropic mediator of growth and survival signals, is significantly reduced or lost in mesenchymal HNSCC tumors [7]. Trop2 consists of a large extracellular domain, a single-pass transmembrane domain, and a twenty-six amino acid tail. Oncogenic signaling can be initiated either by gain- or loss-of-function depending on cell type and context. Ectopic Trop2 expression was shown by our group as well as others to promote tumor growth in NIH3T3 [8] and pancreatic cancer [9] cells, respectively. Other studies indicate a pro-tumorigenic consequence for Trop2 loss. Using a skin carcinogenesis assay in a Trop2 knockout animal, we showed that Trop2 deletion contributes to squamous cell tumorigenesis [7]. More recently, it was reported that the Trop2 gene locus is methylated in a significant fraction of adenocarcinomas of the lung and that this loss contributes to aggressiveness in that disease [10].

The ErbB receptor family plays an important role in HNSCC. Basal subtype tumors are suggested to signal predominantly through epidermal growth factor receptor (EGFR) [2]. Other ErbB family members – ErbB2 and ErbB3 – have also been reported to be commonly expressed in HNSCC, but their significance and relationship to subtype in HNSCC is less well established [11-13]. Although members of this superfamily share basic structural features, each one exhibits unique properties that contextualize their signaling, diversify the range of outputs, and determine patterns of response to therapeutic inhibition [14]. No ligand for ErbB2 has yet to be identified, but the gene is often amplified in breast and gastric cancer and is potently oncogenic in multiple experimental systems [15, 16]. ErbB3 (HER3) is notable as the only EGFR family member that is unable to homodimerize, and it also lacks strong intrinsic kinase activity [17]. As such, it requires dimerization with and transphosphorylation by its preferred partner, ErbB2, but it can also dimerize with ErbB4, or EGFR, upon which ErbB3 potently activates the PI3 kinase pathway [18]. The initiating signals that influence ErbB3 activation are diverse and include post-translational modifications such as translocation from intracellular stores [19]. Recently, ligand-independent mutational activation of ErbB3 has been reported [20], but autocrine signaling via the neuregulin (NRG) family of ErbB3 ligands remains the most well established mechanism of ErbB3 activation [21-23].

Neuregulins, like all EGF family ligands, are generated as transmembrane precursor proteins. Cleavage of the ectodomain is achieved by the ADAMs (A disintegrin and metalloproteinase) family of zinc-dependent membrane-associated metalloproteinases [23, 24], which release the active ligand. ADAM-mediated cleavage can be regulated by oncogenes and other intracellular signals such as protein kinase C [25-27], but additional details of the molecular mechanisms determining neuregulin release are limited. Herein, we report a novel mechanism of NRG1 regulation involving Trop2. NRG1 interacts with Trop2, and when Trop2 is lost, an increase in surface expression of NRG1, NRG1 cleavage, and ErbB3 activation occurs. The relationship between low Trop2 expression and elevated ErbB3 activation was also observed in primary HNSCC tumors, and importantly, we demonstrate that Trop2 loss confers sensitivity to ErbB3 antibodies in tumor xenografts derived from HNSCC cells.

## RESULTS

**Figure 1:**
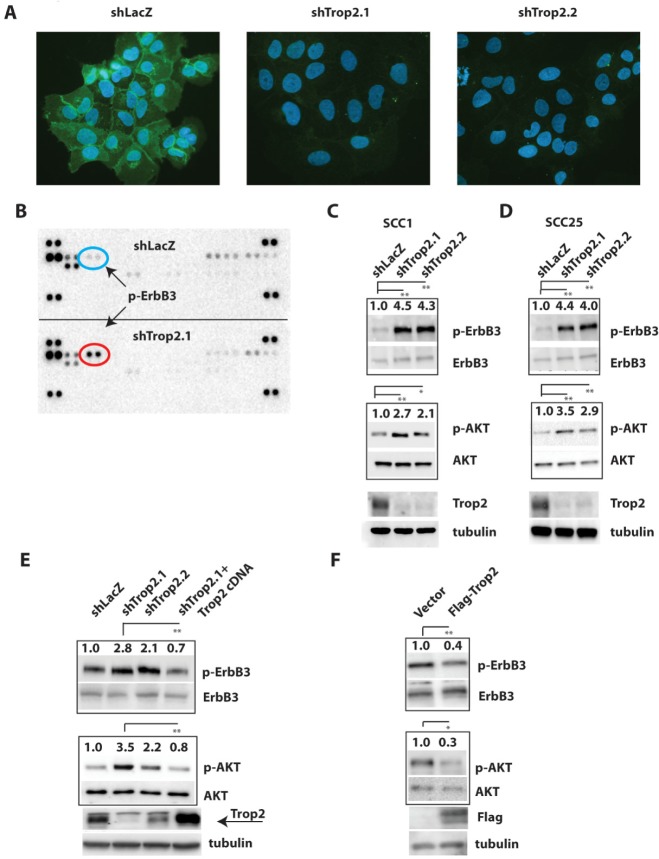
Trop2 Loss Promotes ErbB3 Activation in HNSCC Cells A. Immunofluorescence images of Trop2 staining (green) in SCC1 HNSCC cells after stable knockdown using short hairpins targeting the Trop2 cDNA. Nuclei are counterstained with 4′6-diamidino-2-phenylindole (DAPI). B. Results of a phosphorylated receptor tyrosine kinase antibody array demonstrating elevated p-ErbB3 in lysates from Trop2 knockdown SCC1 cells. The exposures were normalized to the control spots (four corners), which exhibit equal intensities. C&D. Representative immunoblots showing hyperactivation of ErbB3 and AKT caused by Trop2 loss in SCC1 and SCC25 HNSCC cells. Two short hairpins targeting distinct regions of the Trop2 cDNA were used. E. Reduction of ErbB3 activity after ectopic expression of an RNAi-resistant Trop2 cDNA in Trop2 knockdown SCC1 cells. Arrow points to the lower band which is the correct size for Trop2. F. Ectopic expression of a Flag-epitope tagged Trop2 cDNA in SCC1 cells suppresses basal ErbB3 and AKT activation. Control is an empty vector. Relative increases in phosphoproteins in control versus experimental groups were quantified by photodensitometry after normalization to total ErbB3 or AKT protein which served as an internal controls. Immunoblots are representative of at least three independent experiments. Significance was measured by student's *t* test, * (*P*<0.05), ** (*P*<0.01), *** (*P*<0.001).

To investigate the relationship between Trop2 loss and signaling in HNSCC, we examined the effects of reducing Trop2 levels in head and neck squamous cancer cells. Using two short-hairpins targeting unique regions of the Trop2 coding sequence, Trop2 levels were first reduced in the SCC-1 oral squamous cell cancer cell line (Figure 1A&C). As Trop2 resides in large part at the cell surface (in addition to the cytosol), we reasoned that Trop2 is likely to modify signals originating at the plasma membrane and chose to globally interrogate signaling changes caused by Trop2 loss using an unbiased phospho-proteomic approach. Therefore, using an antibody array that simultaneously captures the signaling status of forty-two transmembrane receptor tyrosine kinases, many of which are implicated in tumor biology, we measured the effects of Trop2 loss on phosphorylated (activated) forms of these proteins. Protein lysates from SCC-1 control and knockdown cells were generated and exposed to antibody arrays. Strikingly, the only receptor whose activation was observed to increase upon Trop2 loss was ErbB3 (Figure 1B), a member of the ErbB family implicated in several aspects of aggressive tumor behavior [28-30]. No increase in signaling in any other ErbB family members or other pathways such as IGF-1R was detected after Trop2 loss. Interestingly, the p-Met signal is significantly attenuated after Trop2 loss, possibly due to a negative feedback mechanism (Supplementary Figure 1).

To confirm that Trop2 loss induces ErbB3 activation in HNSCC cells, we measured the levels of p-ErbB3 levels after Trop2 depletion in SCC-1, SCC-25, and Cal 27 cells by immunoblot analysis. Consistent with the result observed on the antibody array, when Trop2 protein expression was reduced, ErbB3 became activated as revealed by Tyr1289 phosphorylation using a phospho-specific antibody (Figure 1C &D, Supplementary Figure 2A). In addition, downstream signaling to AKT was also increased upon Trop2 loss as judged by immunoblots against Ser473 phosphorylation on AKT. To rule out the possibility that the observed Trop2 loss-induced ErbB3 activation was caused by an off-target effect from RNA interference, we further investigated the specific contribution of Trop2 to ErbB3 activity. An RNAi-resistant Trop2 cDNA was engineered by generating silent mutations in the cognate sequence targeted by the vector shTrop2.1. Upon lentiviral transduction of this plasmid into SCC1-shTrop2.1 cells, Trop2 re-expression was able to reduce ErbB3 and AKT activation (Figure 1E). In a parallel experiment, we transduced a Flag-epitope tagged Trop2 cDNA into parental SCC-1 cells and found it capable of suppressing basal levels of p-ErbB3 and subsequent AKT activity (Figure 1F). Collectively, these results suggest that Trop2 can function as an inhibitor of ErbB3 activity.

**Figure 2:**
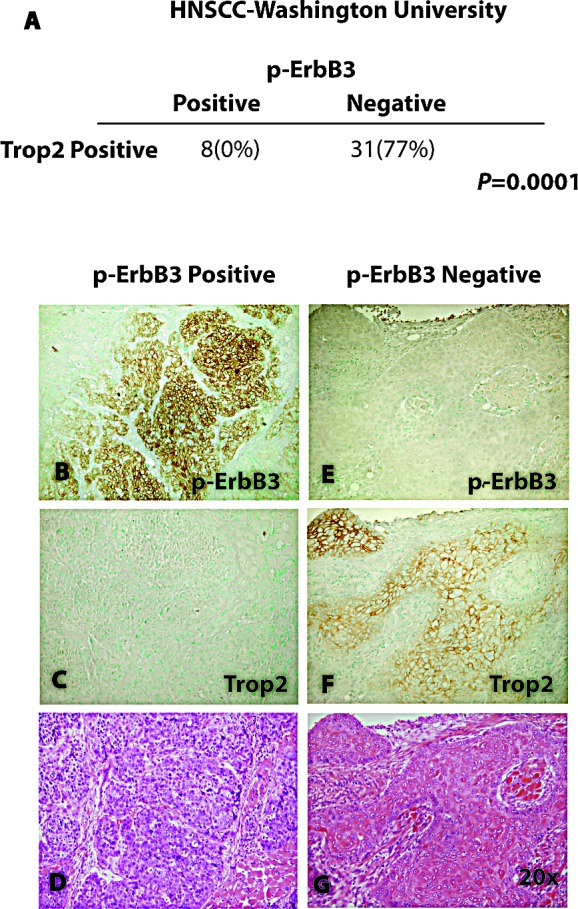
Inverse Correlation Between Trop2 and p-ErbB3 in Primary HNSCC Enumeration of ErbB3 positive or negative tumors as a function of Trop2 staining reveals an inverse correlation significant to *P*=0.0001 (Fisher's exact test). Immunohistochemical staining of a representative squamous cell cancer showing membrane expression of p-ErbB3 (B) and absence of Trop2 expression in an adjacent section (C) in a tumor section showing poorly differentiated squamous histology after hematoxylin and eosin staining (D). Immunohistochemical staining of a representative squamous cell cancer showing absence of p-ErbB3 expression (E) and increased Trop2 expression in an adjacent section (F) in a tumor showing well-differentiated histology after hematoxylin and eosin staining (G). Images are photographed at 20x power.

Next, we sought to determine whether an inverse correlation between Trop2 and NRG1-ErbB3 pathway activation could be identified across tumors from HNSCC patients. Ideally, we would have liked to determine whether NRG1 is cleaved in the absence of Trop2 in tumor samples, but cleavage specific antibodies are not available and the multiple variant isoforms of NRG1 exist further making such an approach ambiguous. Therefore, to judge the relationship between activation of the NRG1-ErbB3 pathway and Trop2 expression, we chose to measure the presence of ErbB3 phosphorylation and correlate this with Trop2 status. We reasoned that tumors that are predominantly Trop2 positive would have minimal evidence of p-ErbB3 expression, and conversely, those with less Trop2 expression would exhibit more p-ErbB3 staining. To address these issues, we obtained thirty-nine freshly cut sections of primary HNSCC and examined multiple regions of each section. P-ErbB3 expression was not observed in any of the thirty-one tumors that showed uniform Trop2 expression. We never observed a signal for p-ErbB3 in any Trop2 positive cells although total ErbB3 expression was found to be comparable in Trop2 positive and negative tumors (Supplementary Figure 3). In contrast, tumors that were Trop2 negative were the only ones to show evidence of p-ErbB3 staining (*P*=0.0001, Figure 2A-G, Supplementary Figure 3). Hence, in accord with the observation that Trop2 loss triggers ErbB3 activation in cell culture models of HNSCC, ErbB3 activation is inversely correlated with loss of Trop2 expression in primary HNSCC.

**Figure 3:**
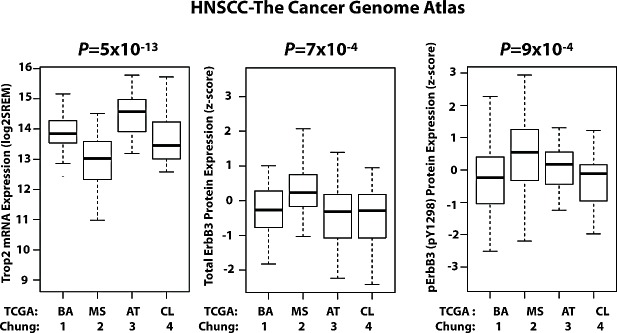
Inverse Correlation Between Trop2 and p-ErbB3 as a Function of HNSCC Subtypes in the TCGA HNSCC Dataset Box and whisker plots of Trop2 expression (left panel) and total and p-ErbB3 protein expression by RPPA (middle and right panels respectively), as functions of Chung and TCGA subtype calls. The data reveal increased total and p-ErbB3 activation in the mesenchymal (group 2, Trop2-low) subtype.

To investigate the relationship between Trop2 and p-ErbB3 in a larger cohort of tumors, we analyzed TCGA dataset. Unfortunately, protein expression for Trop2 is not available, and therefore we correlated Trop2 mRNA expression with total and p-ErbB3 protein expression. We previously reported that Trop2 mRNA expression is reduced in the mesenchymal group of tumors [7], which represented approximately twenty-five percent of the total number of tumors from the Chung dataset [2]. We confirmed the association between low Trop2 mRNA expression and the mesenchymal subtype in TCGA (log-fold change -1.1, *P*= 5×10^−13^), where the mesenchymal subtype represents twenty-four percent of tumors (Figure 3). Next, we analyzed ErbB3 signaling as a function of HNSCC subtype using the TCGA reverse phase protein array (RPPA) data. In this analysis, we found that both total ErbB3 and p-ErbB3 (pY1298) were expressed at higher levels in the mesenchymal subtype compared to the other subtypes (log-fold change 0.6, P=7×10^−4^ for total ErbB3, and log-fold change 0.6, P=9×10^−4^, for p-ErbB3). These data confirm the relationship between low Trop2 expression and enrichment for ErbB3 activity that we initially observed in our functional studies of Trop2 loss.

**Figure 4:**
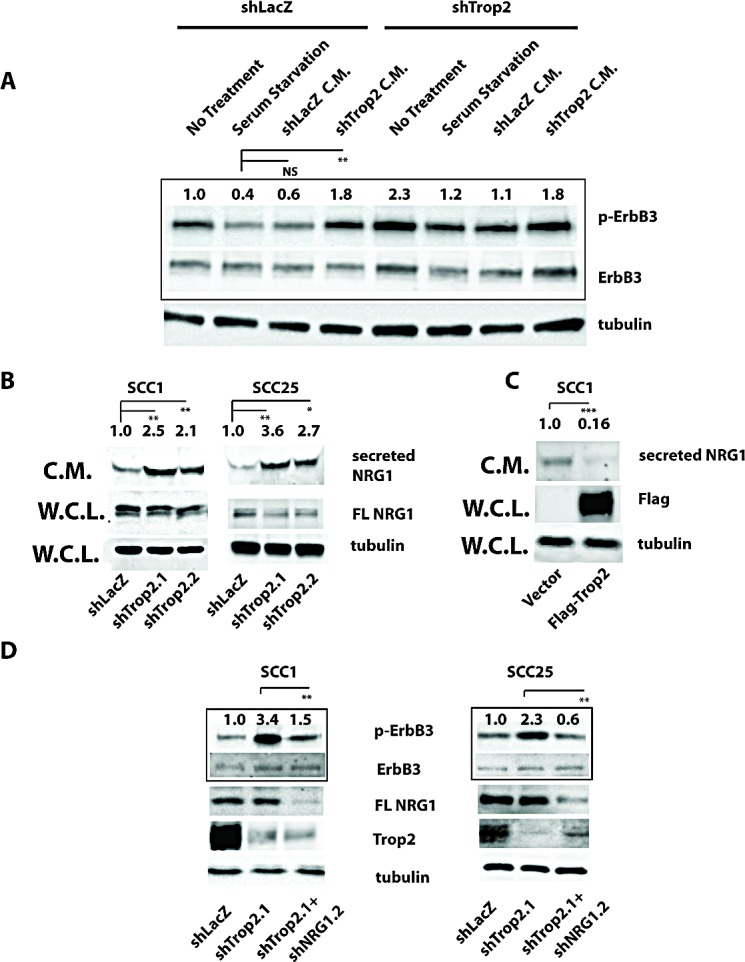
Trop2 Loss Activates ErbB3 Through NRG1 A. Serum-free conditioned media from Trop2 knockdown cells stimulates ErbB3 activity in SCC1 cells. Cells grown in serum-free media for two hours show decreased ErbB3 activity by immunoblot analysis (lanes 1 vs. 2 and 5 vs. 6) that is increased after two hour exposure to conditioned media (C.M.) from Trop2 knockdown cells (shown in lanes 4 and 8) but not by conditioned media from shLacZ control knockdown cells (shown in lanes 3 and 7). Media was conditioned for twenty-four hours prior to harvest. B. Immunoblot analysis of NRG1 protein levels from equal amounts of concentrated serum-free conditioned media or whole cell lysates (W.C.L.) taken from Trop2 knockdown or LacZ control cells. Blots show increased NRG1 secretion but no change in cellular full length NRG1 (FL NRG1) in the absence of Trop-2. Trop2 knockdown lanes are normalized to shLacZ. C. Immunoblot analysis of NRG1 protein levels from equal amounts of concentrated serum-free conditioned media taken from SCC1 cells harboring an empty vector or a Flag epitope-tagged Trop2 cDNA shows reduced soluble NRG1 upon Trop2 expression. Trop2 overexpression lane is normalized to vector control values. D. Immunoblot analysis showing that NRG1 knockdown in Trop2-depleted cells suppresses Trop2-loss induced ErbB3 activation. FL NRG1 is full length NRG1. Significance was measured student's *t* test, * (*P*<0.05), ** (*P*<0.01), *** (*P*<0.001).

To elucidate the mechanism responsible for Trop2-loss induced ErbB3 activation, we first examined whether basal p-ErbB3 levels in control SCC1 cells are sensitive to removal of growth factor. We observed that after two hours of serum starvation, levels of ErbB3 activity were reduced by over one-half in shLacZ control cells (Figure 4A). We then examined whether the heightened ErbB3 activity in Trop2 knockdown cells was also sensitive to growth factor deprivation, and observed a similar level of reduction of p-ErbB3 in these cells as was observed in shLacZ control cells. To further investigate the role of growth factors in Trop2-loss induced p-ErbB3 activation, we asked whether reducing Trop2 levels results in the secretion of an ErbB3-activating ligand that could replace the ability of growth factor-containing serum to stimulate ErbB3 activity in shLacZ control cells. To address this question, we reasoned that shLacZ-bearing control SCC-1 cells, which exhibit a low level of basal ErbB3 activity, could serve as a system to assay for a putative soluble ligand. We generated conditioned media from Trop2 knockdown and control cells by growing cells in serum-free media for twenty-four hours and added this media to the different cell populations that had been serum starved for two hours. Importantly, only conditioned media from Trop2 knockdown cells was able to activate serum-starved shLacZ control cells, indicating release of an ErbB3-stimulating ligand upon Trop2 loss (Figure 4A). Next we sought to determine the identity of the ligand responsible for this activity. As neuregulins are the only ligands known to activate ErbB3 and NRG1 has been reported to be expressed in a subset of HNSCC tumors [12], we considered that this family member would be a likely candidate for regulation by Trop2. We therefore measured the effects of reducing Trop2 levels on soluble and full-length NRG1. Using an antibody that recognizes the amino terminus of NRG1, significantly increased NRG1 ectodomain protein levels were identified in concentrated media taken from Trop2 knockdown SCC1 cells. Similar results were obtained in Trop2 knockdown SCC25 and Cal 27 cells (Figure 4B and Supplementary Figure 2B). Interestingly, we did not observe an increase in levels of full length cellular NRG1 upon Trop2 knockdown despite observing increased NRG1 in the media (Figure 4B), suggesting that Trop2 is regulating NRG1 secretion rather than production or destruction. Conversely, overexpression of a Flag epitope-tagged Trop2 cDNA in SCC1 cells significantly reduced the basal levels of released NRG1 relative to levels in control SCC1 cells harboring an empty vector (Figure 4C). To determine whether NRG1 is required for the increased ErbB3 activity observed upon Trop2 loss, we simultaneously depleted Trop2 and NRG1 in HNSCC cells. We tested five NRG1 short hairpins, but only one, shNRG1.2, significantly reduced NRG1 levels. Using this hairpin, we found that NRG1 depletion significantly reduced p-ErbB3 levels in Trop2 knockdown cells, indicating an essential role for NRG1 in this process (Figure 4D). Collectively, these data point to a role for Trop2 in modulating autocrine ErbB3 activation by titrating the amount of NRG1 that is released from its precursor form.

**Figure 5:**
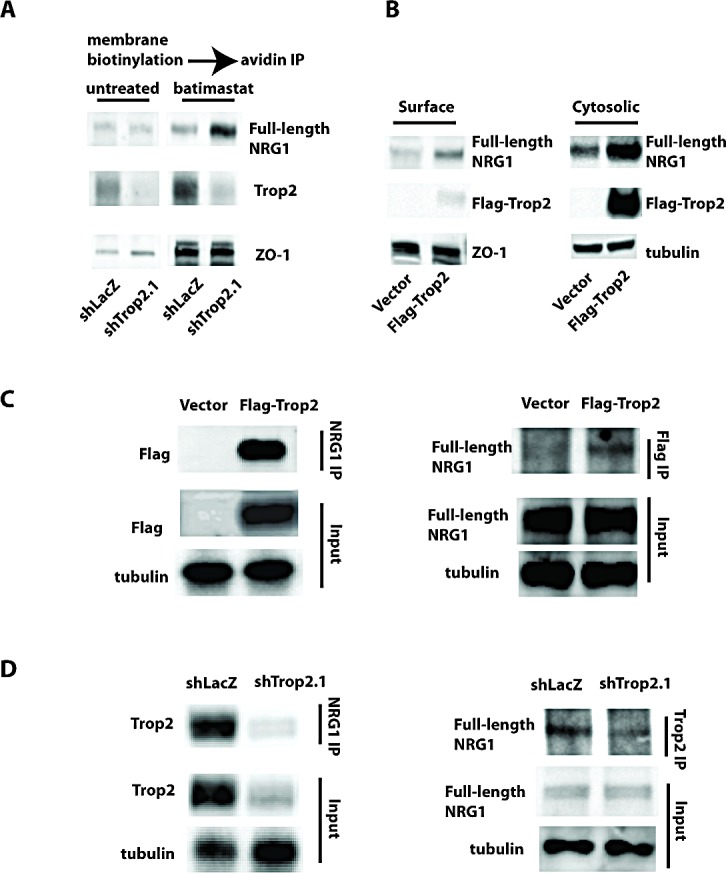
Functional and Physical Interaction Between Trop2 and NRG1 A. Membrane biotinylation and streptavidin immunoprecipitation (IP) reveals an accumulation of cell-surface NRG1 (full length) in Trop2 knockdown SCC1 cells once cleavage is suppressed by batimastat (twenty-four hour treatment). B. Immunblot analysis of biotinylated, streptavidin-precipitated membrane and flow-through cytosolic protein fractions isolated from SCC1 cells after Flag-epitope tagged Trop2 overexpression showing a dramatic increase in cytosolic concentrations of both Trop2 (anti-Flag antibody) and full-length NRG1. C. Immunoprecipitation of NRG1 followed by anti-Flag immunoblot of lysates isolated from cells harboring an empty vector or Flag epitope-tagged Trop2 cDNA reveals an interaction with Trop2 (left panel). The converse experiment (right panel) showing immunoprecipitation using anti-Flag antibodies followed by anti-NRG1 immunblot also reveals the NRG1-Trop2 interaction. D. Immunoprecipitation of NRG1 (left panel) or endogenous Trop2 (right panel) followed by immunoblotting of protein lysates from shLacZ or shTrop2.1 cells using the converse antibodies confirms the NRG1-Trop2 complex. Trop2 knockdown cells serve as a negative control. Results are representative of three independent experiments.

We then considered possible ways by which Trop2 might modulate NRG1 release. Neuregulins localize to cytoplasmic and nuclear pools depending on cell type [31], and are brought to the cell surface and exist as transmembrane precursor proteins prior to cleavage and release of the ectodomain at the plasma membrane. Consistent with these reports, by indirect immunofluorescence staining we found NRG1 to be primarily intracellular in SCC1 cells, visible in both cytosolic and nuclear compartments (Supplementary Figure 4). Given these observations, we investigated whether the absence of Trop2 increases the cell surface concentration of NRG1, which would provide a mechanism of increased NRG1 cleavage and release upon Trop2 loss. To test this possibility, we suppressed NRG1 cleavagewith batimastat, an ADAM17 inhibitor, so as to be able to measure the contribution of Trop2 loss on static levels of NRG1 on the cell surface. After Trop2 depletion in SCC1 cells, we isolated the plasma membrane fraction of cellular proteins by cell-surface biotinylation followed by streptavidin immunoprecipitation, and immunoblotted for NRG1. Strikingly, only in batimastat-treated Trop2 knockdown cells did an increase in membrane-associated, full-length NRG1 become apparent (Figure 5A).

Next, we examined the effects of ectopic Trop2 overexpression on NRG1 localization; Trop2 localizes to both the membrane and cytoplasm (Supplementary Figure 5) and we asked whether increasing Trop2 concentrations in either of these locations would accordingly increase the resident amount of NRG1. We again performed cell-surface biotinylation and streptavidin immunoprecipitation to examine membrane concentrations of Trop2 and NRG1, and also examined the effects of increased Trop2 on the cytoplasmic pools of NRG1 by cytosolic fractionation (Figure 5B). Ectopic Trop2 expression increased Trop2 levels in both in membrane and cytosolic fractions, and NRG1 levels were also comparably elevated in these compartments relative to cells infected with an empty vectorŠ. Taking the effects of both gain- and loss-of function of Trop2 on NRG1 localization into account, these data suggest that Trop2 sequesters NRG1 and may influence its trafficking to or from the cell surface. To develop insight into how this might occur, we sought evidence of a NRG1-Trop2 protein interaction. We immunoprecipitated endogenous soluble NRG1 in Trop2-Flag expressing cells, immunoblotted resolved proteins with anti-Flag antibodies, and observed evidence of NRG1-Trop2 complex formation (Figure 5C). Immunoprecipitation of protein lysates from these cells with the anti-Flag antibody further revealed evidence of a NRG1-Trop2 complex. In addition, when either NRG1 or endogenous Trop2 was immunoprecipitated from cells expressing the control hairpin, the existence of a NRG1-Trop2 complex was also observed, and immunoprecipitations from the knockdown cells served as negative controls (Figure 5D). Lastly, using the RNAi-resistant Trop2 cDNA as a template, we constructed Trop2 mutants harboring a deletion in either the EGF-like or thyroglobulin type-1 domains present in the extracellular region, both of which are required by Trop2 to bind IGF-1 in lung cancer cells [10]. Similar to this requirement, deletion of either domain abolishes the Trop2 interaction with NRG1, and importantly, eliminates the ability of the mutants to suppress ErbB3 activity in Trop2 knockdown cells (Supplementary Figure 7). Collectively, these results indicate that Trop2 is part of a complex with NRG1, and suggest that Trop2 loss increases NRG1 accessibility to the membrane-bound cleavage machinery.

**Figure 6:**
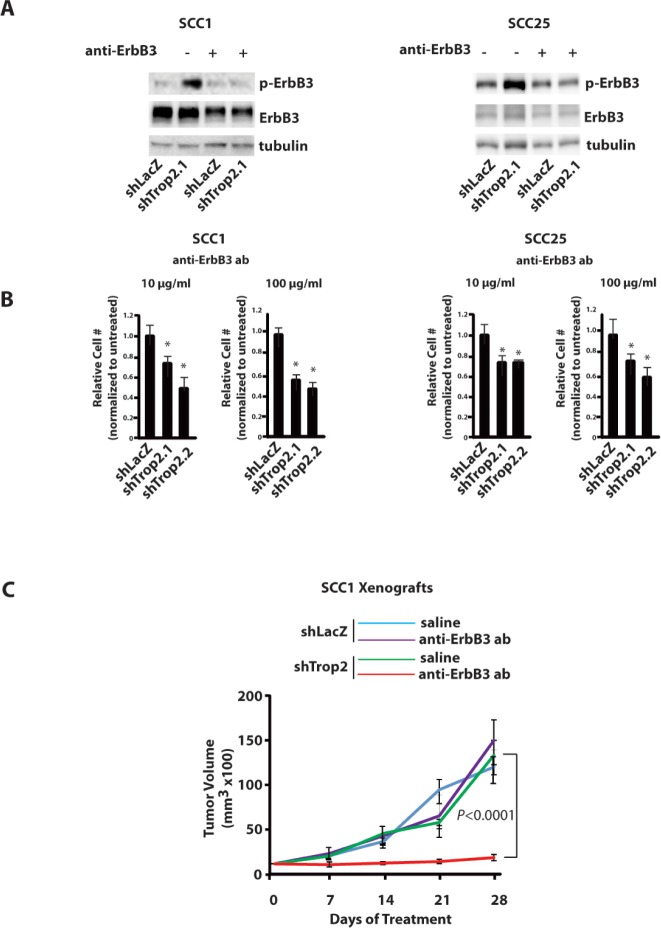
Trop2 Loss Confers Dependency on ErbB3 A. Immunblot analysis of decreased p-ErbB3 in Trop2 knockdown HNSCC cells after four days of treatment with anti-ErbB3 antibodies (DL3.6b). B. Quantification of cell proliferation after seven days of anti-ErbB3 treatment shows reduced proliferation only in Trop2 knockdown cells. Cell proliferation in LacZ controls was unaffected (not shown). Numbers were normalized to untreated cells. Columns mean, bars SE. (*,*P*<0.01 Fisher's exact test). C. The anti-ErbB3 antibody has a specific effect against Trop2-low, ErbB3-activated SCC1 cells. N=5 mice/group. Mice received 25mg/kg antibody weekly for four weeks. *P*<0.0001, ANOVA.

Tumors can become addicted to the oncogenic signaling stimulated by ErbB3 activation [12, 32], a situation reminiscent of the addiction to Ras and other oncogenes that occurs in other cancers. In particular, tumors in which NRG1 triggers ErbB3 signaling have been found to be sensitive to anti-ErbB3 antibody treatment [33]. Based on these observations, we considered that if NRG1 is in fact critical for Trop2-loss induced ErbB3 activation, then interfering with the NRG1-ErbB3 axis should exert an inhibitory effect that is specific to Trop2 knockdown cells. To test this possibility, we measured the effect of treatment with an anti-ErbB3 antibody (a gift from Genentech) on proliferation *in vitro*. Equal numbers of cells were seeded in tissue culture dishes, exposed to treatment with antibody for one week, harvested, and cell numbers assayed. Anti-ErbB3 antibodies can block ligand binding, receptor activation, and induce downregulation [33], and the latter two phenomena were readily detectable in Trop2 knockdown cells treated with the anti-ErbB3 antibody within four days of treatment (Figure 6A). Importantly, the loss of ErbB3 activity in Trop2 depleted cells was associated with a decrease in cell number when enumerated after seven days of treatment (Figure 6B), results that suggested to us that anti-ErbB3 antibodies would also be useful to treat tumors in which Trop2 loss stimulates the NRG1-ErbB3 axis. To determine if this is the case, we performed a proof-of principle experiment by evaluating the effects of treatment with the anti-ErbB3 antibody on tumor xenografts derived from control or Trop2 knockdown SCC-1 cells. Trop2 knockdown cells exhibit a small growth advantage over control cells when grown as tumor xenografts in immunocompromised mice, but this increase is not statistically significant (data not shown). However, when xenografts were allowed to grow to 400 mm^3^ in size prior to weekly antibody treatment, the potent and specific anti-tumor properties of the ErbB3 antibodies against the tumors lacking Trop2 was revealed. These tumors ceased to grow within one week of treatment, and failed to grow for the duration of the four week experiment. In stark contrast, no anti-tumor effect was observed in control cells (Figure 6C). Together, the *in vitro* and *in vivo* data indicate that anti-ErbB3 antibodies are likely to have potent anti-tumor properties against cancers that exhibit low Trop2 and high p-ErbB3 expression.

## DISCUSSION

For numerous tumor types, the identification of specific molecular features that define intrinsic subsets has dramatically improved the ability to predict the efficacy of many targeted therapies and has facilitated the rational development of novel therapeutics. The treatment of *HER2Neu* amplified breast cancer with trastuzumab and b-Raf mutant melanoma with vemurafenib are two examples where subtyping has had a transformative impact [34]. The power of subtyping, whether it is based on genomic or epigenetic signatures, lies in the identification of the underlying biology that drives the subset of cancers, allowing for tailored and targeted treatments. Unfortunately, subtyping in HNSCC has yet to be integrated into the treatment of this disease. The use of cetuximab in HNSCC exemplifies these deficits: its efficacy is limited to a minority of patients and no tumor characteristics have been identified and adopted to select for these patients [35]. Our published analysis of the Chung dataset [7], the currently presented analyses of TCGA dataset, and the results from our immunohistochemical study of primary tumors from our institution, combined with the functional data shown herein point to strong correlations between the mesenchymal subtype, low Trop2 expression, and elevated ErbB3 activity.

**Figure 7:**
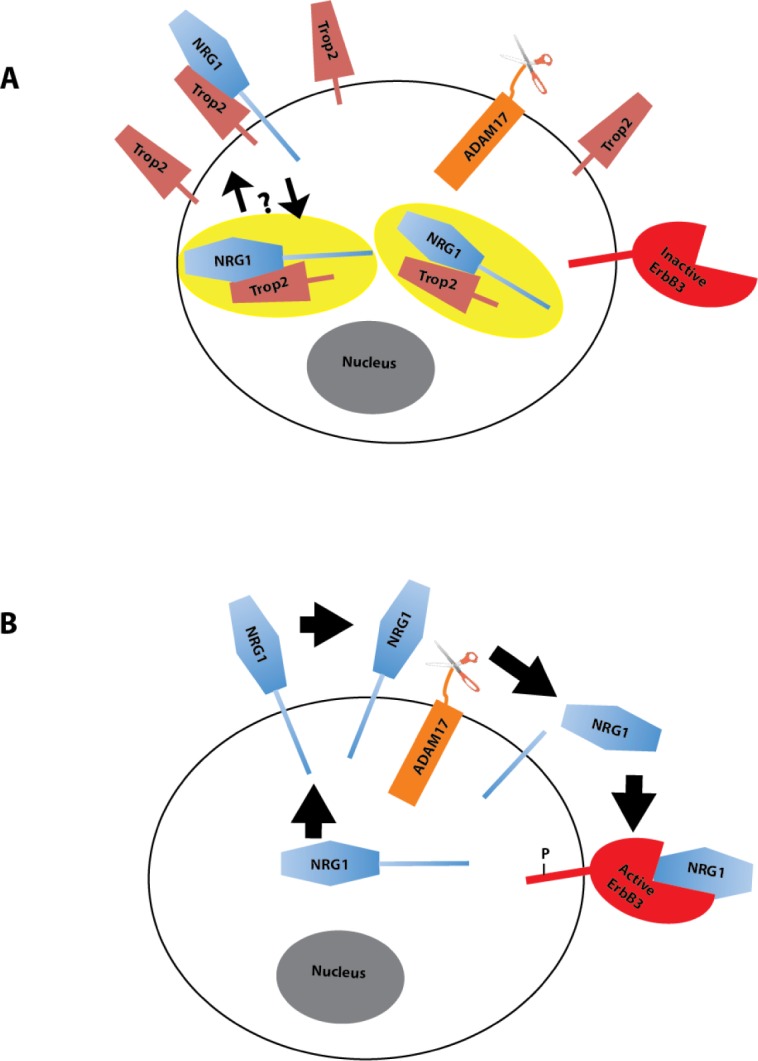
Model of Trop2 Regulation of NRG1 A. NRG1 is predominantly a cytosolic protein and interacts with Trop2, which resides both in the cytosol and membrane pools. It is unknown whether Trop2 and NRG1 interact at the membrane and whether there are pools of the complex that transit to and/or from the membrane. The yellow ellipses represent endosomes. B. Trop2 loss results in a subsequent accumulation of NRG1 at the cell surface where it can be cleaved and released.

Recent studies show that both gain- and loss-of-function of Trop2 can activate oncogenic signaling depending on context [7, 10, 36, 37], however, the ability of Trop2 to influence NRG1 trafficking and release is an unexpected finding. Although modulation of the level of NRG1 cleavage by titration of ADAM17 activity is well established (reviewed in [23, 38]), little is known about how the growth factor itself is regulated independent of ADAM17 activity. Interest in understanding how NRG1 is regulated is growing now that the role of ErbB3 as a mediator of treatment resistance and aggressiveness is becoming widely recognized [19, 39]. One report argues for modulation of NRG1 expression at the level of transcription in some HNSCC tumors [12]. More recently, another layer of regulation, namely phosphorylation of NRG1 by protein kinase C (PKC) prior to cleavage, has been documented [27]. The ability of Trop2 to influence NRG1 trafficking and cleavage now adds another layer of regulation. We note that how Trop2 loss results in increased cell-surface NRG1 is unclear. One possibility, our favored model, is that Trop2 functions as an intracellular retention factor for a distinct pool of NRG1 that is otherwise destined to transit to the cell surface (Figure 7A&B). Alternatively, Trop2 could recycle ambient cell-surface NRG1 such that only in the absence of Trop2 is NRG1 retained at the membrane long enough to be cleaved and released.

The observations reported herein that tumors lacking Trop2 exhibit heightened ErbB3 activity have important clinical implications. The use of ErbB3 pathway inhibitors as anti-cancer therapeutics is an area of active clinical investigation, but the range of susceptible target tumors is unknown. Previous work has demonstrated that ligand-dependent ErbB3 activation renders tumors susceptible to anti-ErbB3 antibodies [33], and our observation that the anti-ErbB3 antibody can suppress both proliferation and tumorigenic growth of ErbB3 activated cells is consistent with these findings. While the relationship between NRG1 expression and outcome in primary HNSCC has not been reported to our knowledge, elevated expression of ErbB3, a possible surrogate for activity, has been associated with a poor prognosis in a variety of cancer types [28-30]. Based on an analysis of NRG1 and ErbB3 mRNA in recurrent HNSCC, post-treatment, late stage disease also appears to be enriched for NRG1-mediated ErbB3 activation [40]; whether Trop2 loss is a mechanism for autocrine activation of ErbB3 in this context remains to be evaluated. Should this be the case, Trop2 could prove to be a useful biomarker for ErbB3 activation and therapeutic stratification in the setting of recurrent HNSCC, an incurable disease for which treatment options are limited. We also note that not all Trop2 negative tumors were found to be positive for p-ErbB3 expression. This observation is could be due to a subgroup of Trop2 negative HNSCC for which ErbB3 signaling is dispensible; also possible is that clinical tumor procurement procedures are not yet optimized for capturing the presence of phospho-proteins which are known to be highly labile.

The mechanisms governing Trop2 expression are also not well understood. We note that Trop2 overexpression has been reported in some tumors, including HNSCC [37, 41]; however, we and others have reported high-level basal expression of Trop2 in normal epithelia of the tonsil and oral cavity, as well as other stratified squamous epithelia [7, 42]. Therefore, the significance of these other findings remains unclear. The recent identification that Trop2 is subjected to regulated intramembrane proteolysis in prostate cells suggests that in some contexts, Trop2 regulation may be a dynamic process [36]. In contrast, Trop2 has been reported to be inactivated by methylation in some lung cancers [10], motivating us to begin to investigate the methylation status of the Trop2 promoter in tumors from TCGA dataset. Ongoing studies such as these are designed to further characterize the molecular details and dependencies arising in the mesenchymal subset of HNSCC.

To conclude, we have identified a direct link between Trop2 and NRG1-ErbB3 signaling in a subset of HNSCC. These data provide a foundation for further functional studies of NRG1 trafficking as well as Trop2 biology. In addition, more extensive genomic and proteomic analyses of Trop2-low HNSCC will likely uncover additional information that will be useful to unravel the molecular basis of aggressive epithelial tumorigenesis. Most importantly, the work described herein delineates a framework for near-term clinical studies of ErbB3 inhibitors against a group of tumors for which these inhibitors are likely to have a high chance of success.

## MATERIALS AND METHODS

### Cell culture

UM-SCC1 (provided by University of Michigan), SCC25, and Cal 27 (both from ATCC) were grown in DMEM/F12 containing 10% FBS. Cell lines were authenticated and matched in February 2013 to short tandem repeat DNA using the Johns Hopkins University Fragment Analysis Facility.

### RNA Interference

Stable short hairpins targeting human Trop-2 cDNA with the following sequences were cloned into the lentiviral vector pLKO-puro (provided by S.A.Stewart, Washington University): GCGCACGCTCATC TATTACCT (nucleotides 1377-1397, ShTrop2.1). CGTGGACAACGATGGCCTCTA (nucleotides 906-926, ShTrop2.2). A short hairpin targeting lacZ was used as a negative control (provided by S.A. Stewart, Washington University in St. Louis). RNAi (1377) resistant hTrop2 sequence is 5′-gcgcacgctgatatactacct, which contains four nucleotides with silent point mutation in RNAi target region using Stratagene Quick Change II Site Mutagenesis Kit. The NRG1 short hairpin sequence is GCCTCAACTGAAGGAGCATAT. Lentivirus infection was performed as described previously [8]. After infection, cells were selected in puromycin.

### Phospho-receptor tyrosine kinase array

The phospho-RTK array (R&D Systems) specifically screens for membrane associated receptors. Antibodies against 42 different RTKs were pre-spotted in duplicate on nitrocellulose membranes. Cell lysates from SCC1 ShLacZ and ShTrop2 cells were incubated with the membrane according to the manufacturer's instructions. Thereafter, a pan anti-phosphotyrosine antibody was used to detect the phosphorylated tyrosine on activated RTKs. The blots were also developed using the ECL method.

### Immunoblotting and Immunoprecipitation

Cells were lysed and homogenized with RIPA lysis buffer with protease inhibitors. Antibodies used in western blotting include: rabbit anti-phospho-AKT (#4060, Ser473, Cell Signaling), rabbit anti-phospho-HER3 (#4791, Tyr1289, Cell Signaling) and mouse or rabbit anti-ErbB3 (Santa Cruz Biotechnology), rabbit anti-NRG1 (Santa Cruz Biotechnology), mouse anti-NRG1 (MAB377, R&D Systems), goat anti-human Trop2 (AF650, R&D Systems), anti-ZO-1 (Life Technologies). The immunoblots were developed using the ECL method (Thermo Scientific).

For immunoprecipitation, cells were scraped off the plates and washed with PBS. Lysis buffer (25mM Tris-Cl, PH 7.5, 225mM NaCl, 5% Glycerol, 1mM EDTA, 25mM NaF, 0.5% NP-40) with protease inhibitor cocktail (Sigma-Aldrich) was used to resuspend the cells. Cell lysates were rocked at 4 ºC for 20 minutes. The insoluble fraction was removed by centrifugation at 12,000g for 5 minutes. 500 μg of total protein was used and anti-NRG1 antibody (MAB377, R&D Systems) or anti-Trop2 antibody (AF650, R&D Systems) was added to the cell lysate for overnight incubation at 4 ºC, then protein G beads were added, and rocked 2-4 hours. 15 μg of protein from the whole cell lysate was resolved and blotted as a loading control. The beads were washed with lysis buffer four times and proteins resolved by SDS-PAGE. Biotinylation was performed according to the manufacturer's instructions (Pierce, Thermo Scientific).

### Immunofluorescence, Immunohistochemistry, and Tissue Samples and Processing

Cells were grown on glass coverslip and washed with PBS, fixed with 4% paraformadelhyde in PBS and permeabilized with 0.1% Triton x-100 in PBS. After several PBS washes, cells were stained for NRG1 (C-20, Santa Cruz Biotechnology) overnight at 4 ºC. Appropriate secondary antibodies were added for one hour at 37 ºC. Cells were mounted and viewed using a Zeiss Axio Plan 2 Fluorescence Microscope. All human samples were collected in accordance with protocols approved by the institutional review board of Washington University Schools of Medicine and data handled confidentially as proscribed.

### *In Vivo* Tumor Xenograft Studies

Athymic nude mice (4 to 6-week-old females) were obtained from Harlan Laboratories (Indianapolis, IN). A suspension of 2×10^6^ SCC1 cells (ShLacZ or ShTrop2) was injected subcutaneously into the right flank of mice. Tumors were measured in length and width once a week and volumes were calculated using the formula (length x width^2^Xπ)/6. All implanted tumors were grown for five to six weeks until average tumor volume reached 400-500 mm^3^ before treatment. At least five mice per group were treated with intraperitoneal injections of 25 mg/kg anti-ErbB3 antibody (Genentech) weekly for five weeks and saline was used a negative control. The change of tumor volumes over time in different groups were described using slopes (i.e., the daily percent increase) and compared by a linear mixed model. The analysis was performed using SAS 9.3 (SAS Institutes, Cary, NC), with a 2-sided p-value of 0.05 to indicate statistical significance.

### TCGA Analysis

Both gene and protein expression were obtained from TCGA using R package cgdsr [43]. Gene expression was measured with RNA-sequencing and all analyses were performed on log transformed, RSEM V2 normalized gene level summaries. Reverse Phase Protein Arrays (RPPA) measured total and phospho protein expression, normalized as z-scores. Intrinsic subtypes were computed from RNA-sequencing using the classifier from [3] as part of the TCGA HNSCC analysis [44]. Differential expression analyses were computed using t-tests comparing expression in the mesenchymal subtype (MS; Chung Subtype 2) to expression in all other subtypes, i.e., Basal (BA; Chung Subtype 1), Atypical (AT; Chung Subtype 3), and Classical (CL; Chung Subtype 4). All analyses were performed on the subset samples reserved for publication for which RPPA measurements were also available as of March 26, 2014.

## SUPPLEMENTAL MATERIAL AND FIGURES



## References

[1] Rothenberg SM, Ellisen LW (2012). The molecular pathogenesis of head and neck squamous cell carcinoma. J Clin Invest.

[2] Chung CH, Parker JS, Karaca G, Wu J, Funkhouser WK, Moore D, Butterfoss D, Xiang D, Zanation A, Yin X, Shockley WW, Weissler MC, Dressler LG, Shores CG, Yarbrough WG, Perou CM (2004). Molecular classification of head and neck squamous cell carcinomas using patterns of gene expression. Cancer Cell.

[3] Walter V, Yin X, Wilkerson MD, Cabanski CR, Zhao N, Du Y, Ang MK, Hayward MC, Salazar AH, Hoadley KA, Fritchie K, Sailey CG, Weissler MC, Shockley WW, Zanation AM, Hackman T (2013). Molecular subtypes in head and neck cancer exhibit distinct patterns of chromosomal gain and loss of canonical cancer genes. PLoS One.

[4] Hayes DN, Grandis J.R., El-Naggar A.K. (2013). The Cancer Genome Atlas: Integrated analysis of genome alterations in squamous cell carcinoma of the head and neck. J Clin Oncol.

[5] Prat A, Perou CM (2011). Deconstructing the molecular portraits of breast cancer. Mol Oncol.

[6] Karapetis CS, Khambata-Ford S, Jonker DJ, O'Callaghan CJ, Tu D, Tebbutt NC, Simes RJ, Chalchal H, Shapiro JD, Robitaille S, Price TJ, Shepherd L, Au HJ, Langer C, Moore MJ, Zalcberg JR (2008). K-ras mutations and benefit from cetuximab in advanced colorectal cancer. N Engl J Med.

[7] Wang J, Zhang K, Grabowska D, Li A, Dong Y, Day R, Humphrey P, Lewis J, Kladney RD, Arbeit JM, Weber JD, Chung CH, Michel LS (2011). Loss of Trop2 promotes carcinogenesis and features of epithelial to mesenchymal transition in squamous cell carcinoma. Mol Cancer Res.

[8] Wang J, Day R, Dong Y, Weintraub SJ, Michel L (2008). Identification of Trop-2 as an oncogene and an attractive therapeutic target in colon cancers. Mol Cancer Ther.

[9] Cubas R, Zhang S, Li M, Chen C, Yao Q (2010). Trop2 expression contributes to tumor pathogenesis by activating the ERK MAPK pathway. Mol Cancer.

[10] Lin JC, Wu YY, Wu JY, Lin TC, Wu CT, Chang YL, Jou YS, Hong TM, Yang PC (2012). TROP2 is epigenetically inactivated and modulates IGF-1R signalling in lung adenocarcinoma. EMBO Mol Med.

[11] Bei R, Budillon A, Masuelli L, Cereda V, Vitolo D, Di Gennaro E, Ripavecchia V, Palumbo C, Ionna F, Losito S, Modesti A, Kraus MH, Muraro R (2004). Frequent overexpression of multiple ErbB receptors by head and neck squamous cell carcinoma contrasts with rare antibody immunity in patients. J Pathol.

[12] Wilson TR, Lee DY, Berry L, Shames DS, Settleman J (2011). Neuregulin-1-mediated autocrine signaling underlies sensitivity to HER2 kinase inhibitors in a subset of human cancers. Cancer Cell.

[13] Xia W, Lau YK, Zhang HZ, Xiao FY, Johnston DA, Liu AR, Li L, Katz RL, Hung MC (1999). Combination of EGFR, HER-2/neu, and HER-3 is a stronger predictor for the outcome of oral squamous cell carcinoma than any individual family members. Clin Cancer Res.

[14] Yarden Y (2001). The EGFR family and its ligands in human cancer. signalling mechanisms and therapeutic opportunities. Eur J Cancer.

[15] Holbro T, Beerli RR, Maurer F, Koziczak M, Barbas CF, Hynes NE (2003). The ErbB2/ErbB3 heterodimer functions as an oncogenic unit: ErbB2 requires ErbB3 to drive breast tumor cell proliferation. Proc Natl Acad Sci U S A.

[16] Muller WJ, Sinn E, Pattengale PK, Wallace R, Leder P (1988). Single-step induction of mammary adenocarcinoma in transgenic mice bearing the activated c-neu oncogene. Cell.

[17] Berger MB, Mendrola JM, Lemmon MA (2004). ErbB3/HER3 does not homodimerize upon neuregulin binding at the cell surface. FEBS Lett.

[18] Amin DN, Campbell MR, Moasser MM (2010). The role of HER3, the unpretentious member of the HER family, in cancer biology and cancer therapeutics. Semin Cell Dev Biol.

[19] Sergina NV, Rausch M, Wang D, Blair J, Hann B, Shokat KM, Moasser MM (2007). Escape from HER-family tyrosine kinase inhibitor therapy by the kinase-inactive HER3. Nature.

[20] Jaiswal BS, Kljavin NM, Stawiski EW, Chan E, Parikh C, Durinck S, Chaudhuri S, Pujara K, Guillory J, Edgar KA, Janakiraman V, Scholz RP, Bowman KK, Lorenzo M, Li H, Wu J (2013). Oncogenic ERBB3 mutations in human cancers. Cancer Cell.

[21] Carraway KL, Sliwkowski MX, Akita R, Platko JV, Guy PM, Nuijens A, Diamonti AJ, Vandlen RL, Cantley LC, Cerione RA (1994). The erbB3 gene product is a receptor for heregulin. J Biol Chem.

[22] Holmes WE, Sliwkowski MX, Akita RW, Henzel WJ, Lee J, Park JW, Yansura D, Abadi N, Raab H, Lewis GD (1992). Identification of heregulin, a specific activator of p185erbB2. Science.

[23] Zhou BB, Peyton M, He B, Liu C, Girard L, Caudler E, Lo Y, Baribaud F, Mikami I, Reguart N, Yang G, Li Y, Yao W, Vaddi K, Gazdar AF, Friedman SM (2006). Targeting ADAM-mediated ligand cleavage to inhibit HER3 and EGFR pathways in non-small cell lung cancer. Cancer Cell.

[24] Blobel CP (2005). ADAMs: key components in EGFR signalling and development. Nat Rev Mol Cell Biol.

[25] Maretzky T, Zhou W, Huang XY, Blobel CP (2011). A transforming Src mutant increases the bioavailability of EGFR ligands via stimulation of the cell-surface metalloproteinase ADAM17. Oncogene.

[26] Van Schaeybroeck S, Kyula JN, Fenton A, Fenning CS, Sasazuki T, Shirasawa S, Longley DB, Johnston PG (2011). Oncogenic Kras promotes chemotherapy-induced growth factor shedding via ADAM17. Cancer Res.

[27] Dang M, Armbruster N, Miller MA, Cermeno E, Hartmann M, Bell GW, Root DE, Lauffenburger DA, Lodish HF, Herrlich A (2013). Regulated ADAM17-dependent EGF family ligand release by substrate-selecting signaling pathways. Proc Natl Acad Sci U S A.

[28] Gilmour LM, Macleod KG, McCaig A, Sewell JM, Gullick WJ, Smyth JF, Langdon SP (2002). Neuregulin expression, function, and signaling in human ovarian cancer cells. Clin Cancer Res.

[29] Yonesaka K, Zejnullahu K, Okamoto I, Satoh T, Cappuzzo F, Souglakos J, Ercan D, Rogers A, Roncalli M, Takeda M, Fujisaka Y, Philips J, Shimizu T, Maenishi O, Cho Y, Sun J (2011). Activation of ERBB2 signaling causes resistance to the EGFR-directed therapeutic antibody cetuximab. Sci Transl Med.

[30] Reschke M, Mihic-Probst D, van der Horst EH, Knyazev P, Wild PJ, Hutterer M, Meyer S, Dummer R, Moch H, Ullrich A (2008). HER3 is a determinant for poor prognosis in melanoma. Clin Cancer Res.

[31] Zhang Z, Prentiss L, Heitzman D, Stahl RC, DiPino F, Carey DJ (2006). Neuregulin isoforms in dorsal root ganglion neurons: effects of the cytoplasmic domain on localization and membrane shedding of Nrg-1 type I. J Neurosci Res.

[32] Sheng Q, Liu X, Fleming E, Yuan K, Piao H, Chen J, Moustafa Z, Thomas RK, Greulich H, Schinzel A, Zaghlul S, Batt D, Ettenberg S, Meyerson M, Schoeberl B, Kung AL (2010). An activated ErbB3/NRG1 autocrine loop supports *in vivo* proliferation in ovarian cancer cells. Cancer Cell.

[33] Schoeberl B, Faber AC, Li D, Liang MC, Crosby K, Onsum M, Burenkova O, Pace E, Walton Z, Nie L, Fulgham A, Song Y, Nielsen UB, Engelman JA, Wong KK (2010). An ErbB3 antibody, MM-121, is active in cancers with ligand-dependent activation. Cancer Res.

[34] Higgins MJ, Baselga J (2011). Targeted therapies for breast cancer. J Clin Invest.

[35] Chen LF, Cohen EE, Grandis JR (2010). New strategies in head and neck cancer: understanding resistance to epidermal growth factor receptor inhibitors. Clin Cancer Res.

[36] Stoyanova T, Goldstein AS, Cai H, Drake JM, Huang J, Witte ON (2012). Regulated proteolysis of Trop2 drives epithelial hyperplasia and stem cell self-renewal via beta-catenin signaling. Genes Dev.

[37] Nakanishi H, Taccioli C, Palatini J, Fernandez-Cymering C, Cui R, Kim T, Volinia S, Croce CM (2013). Loss of miR-125b-1 contributes to head and neck cancer development by dysregulating TACSTD2 and MAPK pathway. Oncogene.

[38] Murphy G (2009). Regulation of the proteolytic disintegrin metalloproteinases, the ‘Sheddases’. Semin Cell Dev Biol.

[39] Engelman JA, Zejnullahu K, Mitsudomi T, Song Y, Hyland C, Park JO, Lindeman N, Gale CM, Zhao X, Christensen J, Kosaka T, Holmes AJ, Rogers AM, Cappuzzo F, Mok T, Lee C (2007). MET amplification leads to gefitinib resistance in lung cancer by activating ERBB3 signaling. Science.

[40] Shames DS, Carbon J, Walter K, Jubb AM, Kozlowski C, Januario T, AnDo, Fu L, Xiao Y, Raja R, Jiang B, Malekafzali A, Stern H, Settleman J, Wilson TR, Hampton GM (2013). High heregulin expression is associated with activated HER3 and may define an actionable biomarker in patients with squamous cell carcinomas of the head and neck. PLoS One.

[41] Fong D, Spizzo G, Gostner JM, Gastl G, Moser P, Krammel C, Gerhard S, Rasse M, Laimer K (2008). TROP2: a novel prognostic marker in squamous cell carcinoma of the oral cavity. Mod Pathol.

[42] Stepan LP, Trueblood ES, Hale K, Babcook J, Borges L, Sutherland CL (2011). Expression of Trop2 cell surface glycoprotein in normal and tumor tissues: potential implications as a cancer therapeutic target. J Histochem Cytochem.

[43] Gao J, Aksoy BA, Dogrusoz U, Dresdner G, Gross B, Sumer SO, Sun Y, Jacobsen A, Sinha R, Larsson E, Cerami E, Sander C, Schultz N (2013). Integrative analysis of complex cancer genomics and clinical profiles using the cBioPortal. Sci Signal.

[44] TCGA https://tcga-data.nci.nih.gov.

