# Supplementary material for: Loss of Trop2 causes ErbB3 activation through a neuregulin-1-dependent mechanism in the mesenchymal subtype of HNSCC
Source: Oncotarget. 2014 Sep 2;5(19):9281–94. doi: 10.18632/oncotarget.2423 (PMC4253434; doi:10.18632/oncotarget.2423)
Supplement: Supplementary file 1 [file oncotarget-05-9281-s001.pdf]

Loss of Trop2 causes ErbB3 activation through a neuregulin-1-dependent mechanism in the mesenchymal subtype of HNSCC

Supplemental Material

Supplementary Figure 1

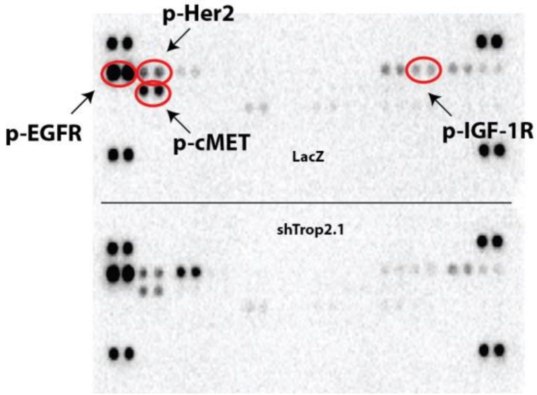

Figure 1: R&D Antibody array showing the effects of Trop2 loss on EGFR, Her2, c-MET, and IGF-1R activity. No measureable increase in the activity of these proteins upon Trop2 loss was observed in this assay.

Supplementary Figure 2

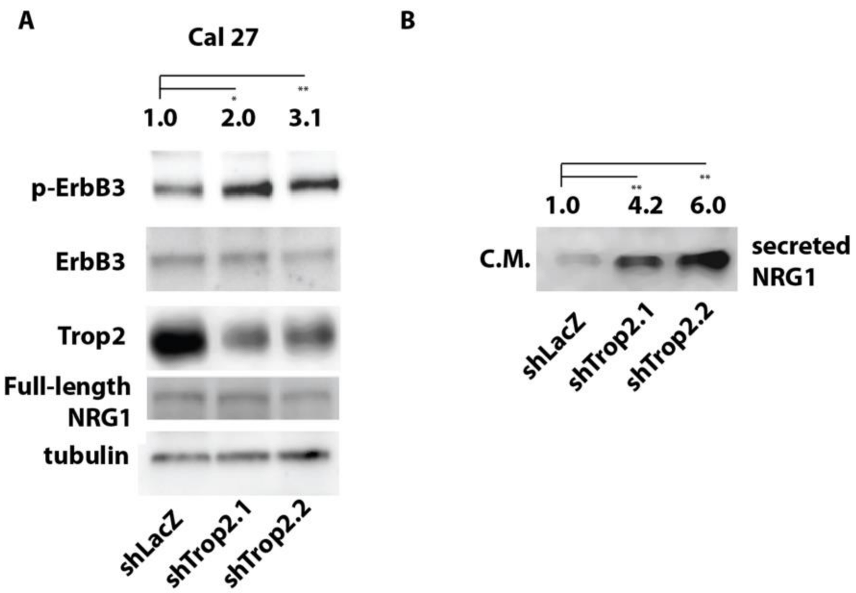

Figure 2: Trop2 loss in Cal 27 cells results in autocrine ErbB3 activation. A. Trop2 knockdown results in hyperactivation of ErbB3. Densitometry images are normalized to total ErbB3. No change in full-length cellular NRG1 is present. B. Concentrated serum-free media taken from Cal 27 cells after a twenty-four hour incubation period (conditioned media, C.M.) reveals increased NRG1 secretion similar to SCC1 and SCC25 cells. Densitometry values are normalized to NRG1 secreted by shLacZ control cells. Results are representative of three independent experiments. Significance was measured student's *t* test, * ($P<0.05$), ** ($P<0.01$), *** ($P<0.001$).

Supplementary Figure 3

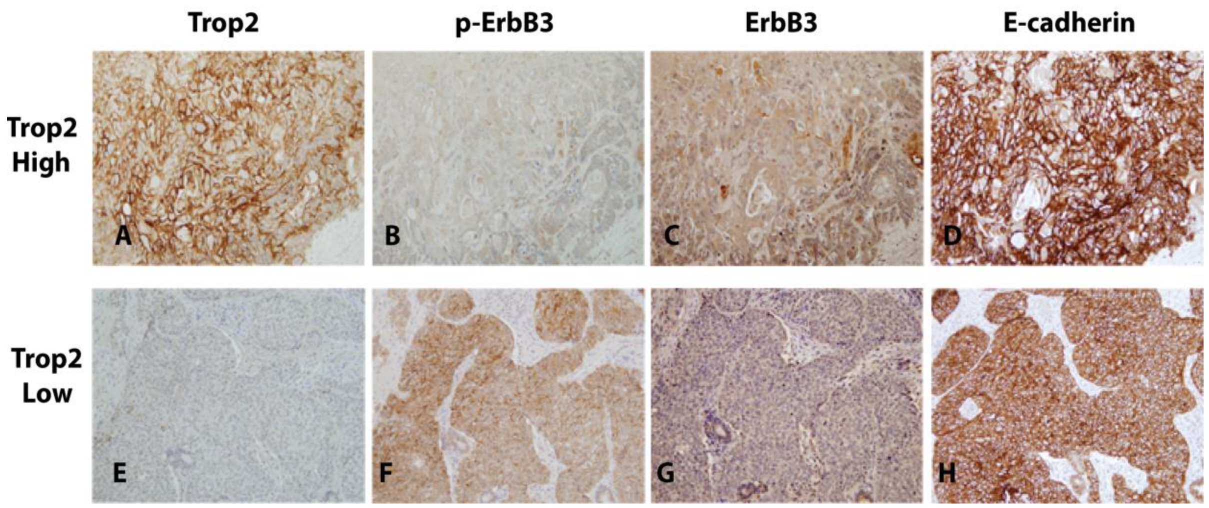

Figure 3: Trop2 expression inversely correlates with ErB3 activation (p-ErbB3 staining). Additional representative cases of primary tumors with high and low Trop2 expression are shown with the corresponding levels of p-ErbB3, ErbB3, and E-cadherin. Panels A-D and E-H are adjacent sections of Trop2 high and low tumors respectively. ErbB3 expression is readily apparent in Trop2 high cases despite the absence of p-ErbB3 staining in these tumors. Images are photographed at 20x power.

Supplementary Figure 4

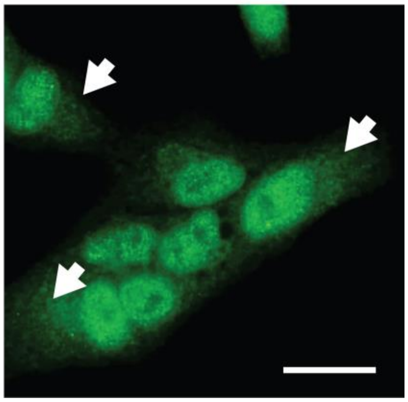

Figure 4: NRG1 localization in HNSCC cells. Indirect immunofluorescence of parental SCC1 cells using a C-terminal antibody against NRG1. Images showing predominant cytoplasmic (white arrows) or nuclear staining. Scale bar: 10 μm.

Supplementary Figure 5

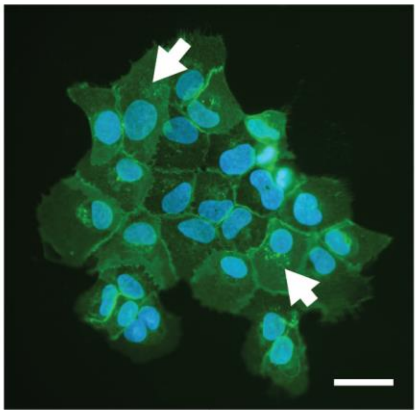

Figure 5: Trop2 localization in HNSCC cells. Indirect immunofluorescence of parental SCC1 using an anti-Trop2 antibody. Cells show cytoplasmic (white arrows) and membrane staining of endogenous Trop2. Scale bar: 10 μm.

Supplementary Figure 6

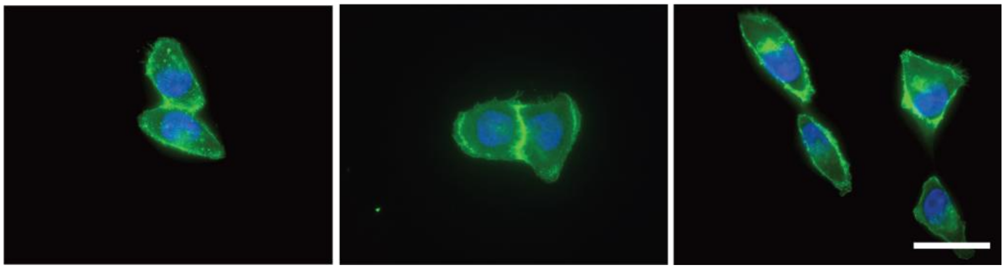

Figure 6: Trop2 localization in sparsely plated HNSCC cells. Indirect immunofluorescence of parental SCC1 using an anti-Trop2 antibody shows retention of cell surface localization in the absence of cell-cell contact. Scale bar: 10 μm.

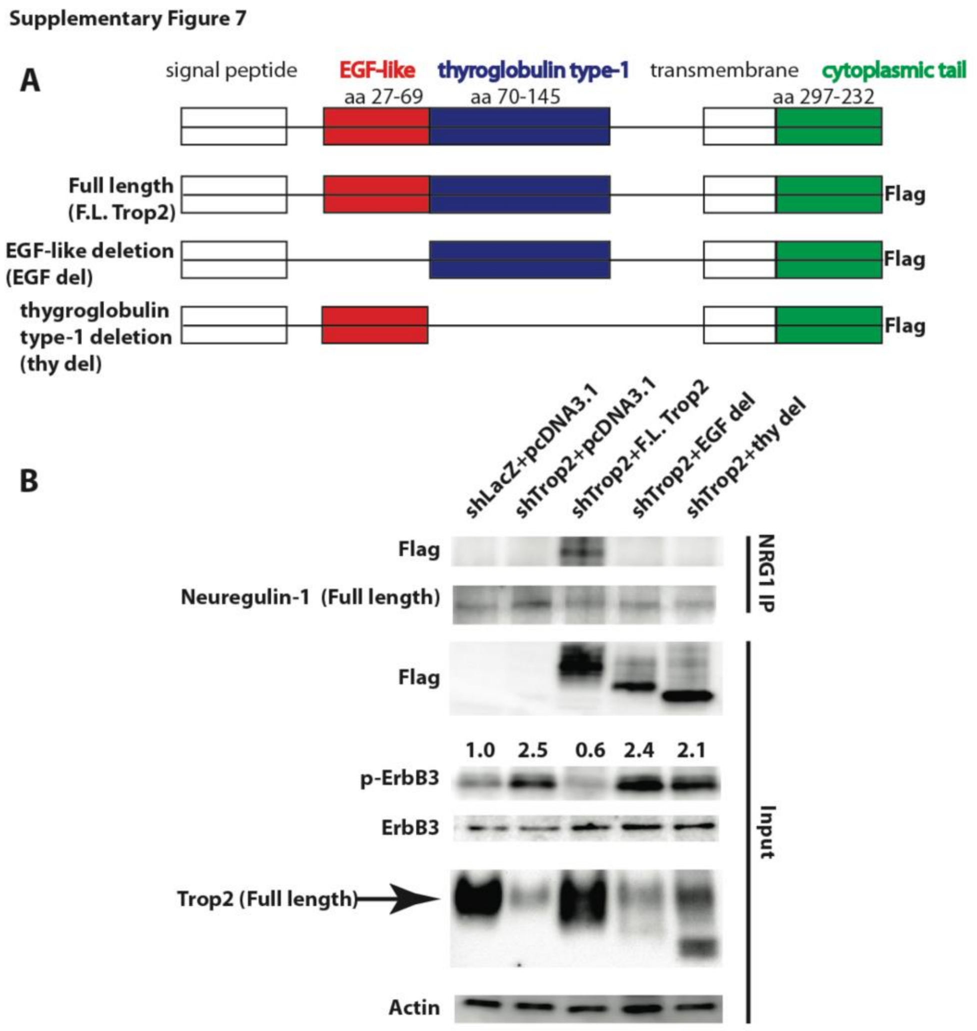

Figure 7: Trop2 suppression of ErbB3 activation requires NRG1 binding by Trop2. A. Diagram of the Trop2 expression constructs cloned into pcDNA3.1, all of which are resistant to the Trop2 short hairpin. These were used to determine the Trop2 domains required to interact with NRG1 and needed to suppress ErbB3 activation. Shown are the full length Flag-epitope tagged Trop2**,** and Trop2 deletion mutants that lack either the EGF-like domain or the thyroglobulin type-1 domain, both of which are found in the extracellular region of Trop2. B. SCC1 cells (stably harboring shLacZ or shTrop2 lentiviral plasmids) were harvested twenty-four hours after being transiently transfected with an empty pcDNA3.1 vector or the specified cDNA inserts. Protein lysates from these cells were used for NRG1 immunoprecipitation (NRG1 IP) and assessment of ErbB3 activation (input). Representative immunoblots of NRG1 immunoprecipitation with anti-mouse NRG1 followed by immunoblotting using anti-Flag antibody or anti-rabbit NRG1 reveal that only the full-length Trop2 cDNA interacts with NRG1. Immunoblots performed on input lysates show that only the full-length Trop2 cDNA can suppress ErbB3 activity. Quantification shows normalization of p-ErbB3 to total ErbB3. The Flag antibody identifies the faster migrating Trop2 deletion mutants. The anti-Trop2 antibody was raised against and recognizes epitopes in the wild-type extracellular domain and is less reactive with the deletion mutants.

Supplementary methods

We made two deletion constructs and one full length Trop construct as indicated. EGF-like domain (amino acids 27-69) or thyroglobulin type-1 domain (amino acids 70-145) deletions were generated by overlap extension PCR using the Trop2 knockdown-resistant (referred to as RNAi-resistant), full-length Trop2 cDNA containing the Flag epitope at the C-terminus as the template. These fragments were initially subcloned into the pSP72 vector, and then transferred back into pcDNA3.1. Overlap extension PCR primers for these two deletion constructs are: 5'-cagcagacacttggaggtgccggtcaccgccgccag and 5'- ctggcggcggtgaccggcacctccaagtgtctgctg for the EGF-like domain deletion and 5'-ggtgcgcac cagctcatccagcgtggagcagtcg and 5'-cgactgctccacgctggatgagctggtgcgcacc for Thyroglobulin type-1 domain deletion. The reverse primer contains the Flag epitope: 5'- cgaattcctatttatcgtcatcgtctttgtagtcgatcaccaggacggcc.
